# Diagnostic Value of Fractional Exhaled Nitric Oxide and Small Airway Function in Differentiating Cough-Variant Asthma from Typical Asthma

**DOI:** 10.1155/2021/9954411

**Published:** 2021-08-20

**Authors:** Yanqi Wang, Lixuan Zhao, Fang Chen, Yufeng Guo, Hongxia Ma, Baofen Han, Jiang Yi, Xiaomei Kong

**Affiliations:** ^1^Shanxi Medical University, Taiyuan, Shanxi 030001, China; ^2^Department of Respiratory and Critical Care Medicine, The First Hospital of Shanxi Medical University, Taiyuan, Shanxi 030001, China

## Abstract

**Purpose:**

To explore the diagnostic value of fractional exhaled nitric oxide (FeNO), small airway function, and a combined of both in differentiating cough-variant asthma (CVA) from typical asthma (TA).

**Methods:**

A total of 206 asthma subjects, including 104 CVA and 102 TA, were tested for pulmonary function, bronchial provocation test and FeNO. The correlation between FeNO, small airway function and other pulmonary indicators was analyzed by single correlation and multiple regression analysis. The receiver operating characteristic (ROC) curve was established to evaluate the diagnostic efficiency of FeNO, small airway function, and their combination and to predict the optimal cut-off point.

**Results:**

All the respiratory function parameters and small airway function indicators in TA group were significantly different from those in CVA group, and FeNO value was significantly higher than that in CVA group. In addition, the area under the ROC curve (AUC) was estimated to be 0.660 for FeNO, 0.895 for MMEF_75%/25%_, 0.873 for FEF_50%_, 0.898 for FEF_25%_, 0.695 for Fres, 0.650 for R5-R20, and 0.645 for X5. The optimal cut-off points of FeNO, MMEF_75%/25%_, FEF_50%_, FEF_25%_, Fres, R5-R20 and X5, were 48.50 ppb, 60.02%, 63.46%, 45.26%, 16.63 Hz, 0.38 kPa·L^−1^·s^−1^, and −1.32, respectively. And the AUC of FeNO combined with small airway function indexes FEF_25%_, Fres, R5-R20, and X5 were prior than single indicators.

**Conclusion:**

FeNO and small airway function indexes might have great diagnostic value for differentiating CVA from TA. The combination of FeNO and FEF_25%_, Fres, R5-R20, and X5 provided a significantly better prediction than either alone.

## 1. Introduction

Asthma is a common respiratory disease. Airway hyperresponsiveness, airway inflammation and reversible airflow limitation are the main pathophysiological characteristics of asthma [1]. Cough-variant asthma (CVA) is a subtype of asthma with chronic cough as a single clinical symptom, without wheezing or dyspnea [2]. Typical asthma (TA) is characterized by recurrent wheezing, chest tightness, or cough, often accompanied by reversible airflow limitation, airway hyperresponsiveness, and airway remodeling [3]. As nearly 30% of CVA will have wheezing, shortness of breath, and other symptoms as the disease progresses and eventually develop into TA, CVA is considered to be the precursor of TA [4]. However, the evolution from CVA to TA has not been fully elucidated. Studies believe that the pathogenesis of CVA is similar to that of TA, which is chronic airway inflammation involving multiple cells [5], and airway inflammation is one of the most common factors that aggravate BHR and cough receptor hypersensitivity [6]. Therefore, it is speculated that CVA will be aggravated to TA due to airway inflammation. In addition, in terms of cell infiltration and inflammatory factor gene expression, TA and CVA seem to have a common inflammation mode [7]. CVA may also have BHR-related persistent coughing with hidden bronchoconstriction without wheezing. The development of CVA to TA is also considered a natural process [8]. Therefore, early diagnosis of CVA is very important, and distinguishing CVA from TA may lead to understanding incipiency of the disease. However, CVA diagnosis remains difficult because chronic, nonproductive cough has a wide range of etiologies.

The bronchial provocation test (BPT) is a key method for the diagnosis of CVA, and the diagnosis of CVA should be based on laboratory evidence of bronchial hyperresponsiveness (BHR) and/or therapeutic effects according to the guidelines [9]. However, BHR detection has some risks and limitations in clinical application [10]. Therefore, it is very important to find a new method for the early diagnosis of CVA and distinguish CVA from TA. In recent years, fractional exhaled nitric oxide (FeNO) and small airway function have been widely used in clinical practice due to their noninvasive, simple, and highly repetitive characteristics in evaluating airway inflammation, or airway reactivity [11].

Nitric oxide (NO) is catalyzed by inducible nitric oxide synthase (iNOS) in airway epithelial cells and produced by L-arginine transformation. FeNO is significantly associated with eosinophilic airway inflammation and plays an important role in the cause of chronic cough [12]. In addition, chronic airway inflammation is the essence of asthma, and the number of inflammatory cells in the small airway is significantly higher than that in the air duct and alveolar tissue during its development [13]. Studies have found that small airway function associates with airway reactivity, control level, and severity of asthma [14]. However, it is not clear whether FeNO combined with small airway function can improve the diagnostic value in differentiating CVA from TA. Therefore, the purpose of this study was to explore the clinical value of FeNO combined with small airways function in differentiating CVA and TA by analyzing TA and CVA patients.

## 2. Materials and Methods

### 2.1. Study Population

A total of 206 subjects in the outpatient service of respiratory and critical care medicine of the First Hospital of Shanxi Medical University from October 2018 to October 2019 participated in the research. The inclusive criteria were as follows: (1) the diagnoses of asthma were in line with the diagnostic criteria in the guidelines for the Global Initiative for Asthma guidelines and Chinese National Guidelines on diagnosis and management of cough: consensus and controversy [15, 16], first diagnosed with asthma; (2) >18 years old; (3) without abnormalities on chest X-ray; (4) without treatment of any oral corticosteroid in the last 4 weeks and without respiratory tract infection within 8 weeks; (5) nonsmokers or ex-smokers with cessation of smoking for at least 6 months prior to the study; and (6) no history of other lung diseases, including but not limited to obliterative bronchiolitis, bronchiectasis, and cystic fibrosis. The study was approved by the Ethical Committee of the First Hospital of Shanxi Medical University. All patients were informed, and their informed consent was collected.

### 2.2. Detection of FeNO

FeNO was measured using a Ncoulomb expiratory analyzer (Sunvou-CA2122, China) and was performed according to the product operation instructions and American Thoracic Society/European Respiratory Society (ATS/ERS) recommendations [17]. Subjects were informed to deeply inhale NO-free air and immediately exhale in full via a mouthpiece at a constant flow rate (50 mL/s) for 10 s. This test was scheduled prior to pulmonary function examination.

### 2.3. Detection of Pulmonary Function

The determination of pulmonary function was performed using Master Screen IOS pulmonary function instrument (Jaeger Co, Germany) in strict accordance with the guidelines of ATS/ERS [18]. The pulmonary function tested in this study included respiratory function (forced expiratory volume in 1 s (FEV1%), forced vital capacity (FVC), FEV1/FVC, FEV1/VC_max_, peak expiratory flow (PEF), forced expiratory flow between 25% and 75% (MMEF_75%/25%_), forced expiratory flow at 50% of the FVC (FEF_50%_), forced expiratory flow at 25% of the FVC (FEF_25%_), total lung capacity (TLC), residual volume (RV), RV/TLC), diffusion function (D_L_CO), impulse oscillometry indexes (Fres, Z5, R5, R20, R5-R20, X5, andX20), and bronchial provocation test (PD20).

### 2.4. Bronchial Provocation Test (BPT)

The acetylcholine was used as the provocative in BPT according to the criteria of the American Thoracic Society [19]. Subjects were asked to inhale the acetylcholine aerosol with a handheld sprayer (PARI GmbH, Germany). Isotonic saline was inhaled first as a control. This was followed by progressive doubling of concentrations of acetylcholine from low to high and the operating air flow rate was 5 L/min. The test was continued until the respiratory resistance increased to two or more times of the base level or the FEV1 had fallen >20% or the maximal concentration of acetylcholine had been administered. The cumulative dose of acetylcholine inhaled when FEV1% decreased by 20% (PD20) was used as quantitative index.

### 2.5. Statistical Analysis

SPSS 20 statistical software was used to analysis the data. The Kolmogorov–Smirnov normality test was performed to check whether continuous variables were normality distributed. Data consistent with normal distribution were expressed as mean ± standard deviation while median (interquartile range) was used for nonnormal distribution. The two-tailed Student's *T*-test and Mann–Whitney *U*-tests were used to compare the differences between the two groups, while frequencies and chi-square test were used to describe the distribution of categorical variables. Pearson correlation was used for normal distribution data, and Spearman correlation analysis was used for nonnormal distribution data to determine data correlation. And the receiver operating characteristic (ROC) curve was used to analyze the diagnostic value of statistically significant indicators and combined indicators for CVA, and to calculate the optimal cut-off point. *P* < 0.05 was considered statistically significant.

## 3. Results

### 3.1. The Characteristics of TA and CVA Subjects

A total of 206 subjects were included in this study, including 102 subjects with TA and 104 subjects with CVA according to the TA and CVA diagnostic criteria. All subjects were given appropriate treatment by the attending physician according to their condition, with medication as the main treatment, and all treatments were aimed at controlling the asthma condition. The asthma control test (ACT) was used to evaluate the level of asthma control in each subject after treatment. The asthma levels of most subjects were well controlled (ACT > 19), which the proportion of TA well controlled was 79.6%, and the proportion of CVA well controlled was 80.1%. There was no significant difference between the two groups. The basic characteristics of the subjects are shown in Table 1. There was a significant difference in gender and age distribution between the two groups (*P* < 0.001). TA subjects were generally older than CVA subjects and mainly distributed in middle-aged and elderly people (>45), while VCA subjects are evenly distributed in young people and middle-aged and elderly people. Moreover, there was no significant difference in BMI between TA and CVA subjects (*P*=0.56).

### 3.2. Comparison of Pulmonary Function between TA and CVA Subjects

**Figure 1:**
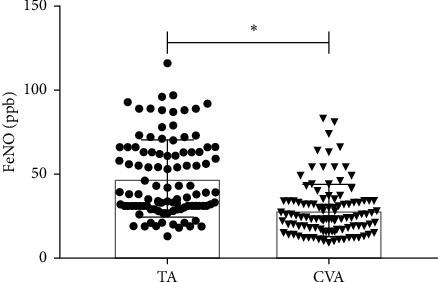
Comparison of FeNO level between the TA and CVA subjects, ^*∗*^*P* < 0.05, compare with TA.

There were significant differences in pulmonary function indicators between TA and CVA subjects. All respiratory function parameters, including FEV_1_%, FVC, FEV_1_/FVC, FEV1/VC_max_, PEF, D_L_CO, and TLC were significantly lower (*P* < 0.001) in the TA subjects than CVA subjects (Table 2). Also, the PD20 was significantly lower in the TA subjects than CVA subjects (*P* < 0.001). In addition, the MMEF_75%/25%_, FEF_50%_, and FEF_25%_, which were related to the measurement of small airway velocity, were significantly higher in CVA subjects than TA subjects. Impulse oscillometry indexes Z5, Fres, and R5-R20 in TA subjects were significantly higher than those in CVA subjects, while X5 and X20 in TA subjects were significantly lower than those in CVA subjects (*P* < 0.01). The FeNO value of TA subjects was significantly higher than that of CVA subjects (*P*=0.007, Figure 1).

### 3.3. Correlations between FeNO or Small Airways Function and Other Parameters

Single correlations and multiple regression analysis of FeNO or small airways function (MMEF_75%/25%_, FEF_50%_, FEF_25%_) and the characteristics factors (sex, age, etc), pulmonary function (FEV1_%_/FVC, etc), and BHR (PD20) were shown in Table 3. Single correlation and multiple regression analysis clarified the independent significant correlations of MMEF_75%/25%_ with FEV_1_/FVC, PEF, DLCO, and RV/TLC in TA subjects (*P* < 0.01) and that with FEV_1_/FVC, PEF, and RV/TLC in CVA, and the significant correlation of FEF_50%_ with FEV_1_/FVC, DLCO, and RV/TLC in TA subjects and with FEV_1_/FVC and RV/TLC in CVA subjects, and the significant correlation of FEF_25%_ with FEV_1_/FVC, DLCO, and RV/TLC in TA subjects and with FEV_1_/FVC in CVA subjects. It is worth noting that in univariate analysis, there was no significant correlation between small airway function and gender or BMI, but in multivariate analysis, MMEF_75%/25%_, FEF_50%_, and FEF_25%_ in TA were significantly correlated with gender and BMI, while MMEF_75%/25%_ and FEF_25%_ were significantly correlated with gender in CVA. To some extent, gender is related to small airway function both in TA and CVA.

### 3.4. ROC Curves of FeNO and Small Airways Function for CVA Subjects

**Figure 2:**
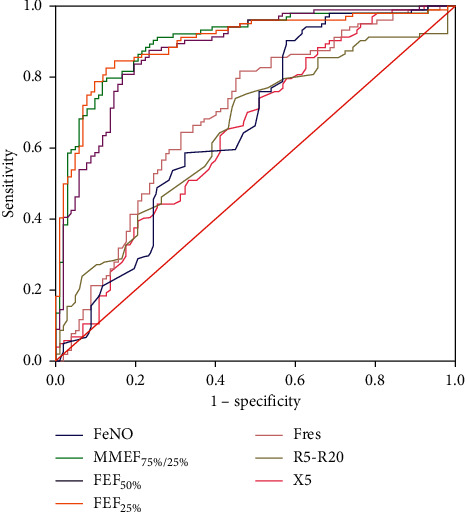
ROC curve of FeNO and small airways function indicators for CVA diagnosis.

**Figure 3:**
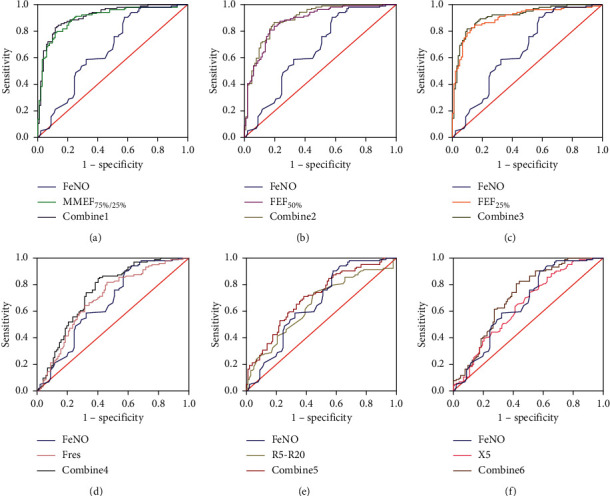
ROC curves for FeNO combined with small airways function in predicting CVA: (a) ROC curves for FeNO combined with MMEF_75%/25%_, (b) ROC curves for FeNO combined with FEF_50%_, (c) ROC curves for FeNO combined with FEF_25%_, (d) ROC curves for FeNO combined with Fres, (e) ROC curves for FeNO combined with R5-R20, (f) ROC curves for FeNO combined with X5. Combine 1: FeNO + MMEF_75%/25%_, Combine 2: FeNO + FEF_50%_, Combine 3: FeNO + FEF_25%_, Combine 4: FeNO + Fres, Combine 5: FeNO + R5-R20, and Combine 6: FeNO + X5.

Significant differences of FeNO and small airways function were observed between the CVA and TA subjects, which may be potential diagnostic indicators for differentiating CVA from TA. To distinguish CVA and TA, the ROC curve (Figure 2) for these parameters were constructed to define the optimal cut-off value for the level of FeNO and small airways function. The area under the ROC curve of FeNO, MMEF_75%/25%_, FEF_50%_, FEF_25%_, Fres, R5-R20, and X5 were 0.660 (0.585–0.735), 0.895 (0.850–0.939), 0.873 (0.825–0.921), 0.898 (0.854–0.942), 0.695 (0.623–0.767), 0.650 (0.575–0.725), and 0.645 (0.570–0.720), respectively (Table 4). The sensitivity and specificity of FeNO in detecting CVA from TA were 90.4% and 42.2% at a cut-off point of 48.50 ppb, while the sensitivity of MMEF_75%/25%_, FEF_50%_, FEF_25%_, Fres, R5-R20, and X5 were 78.8%, 80.8%, 84.6%, 81.7%, 74.0%, and 74.0%, and specificity were 87.3%, 82.4%, 85.3%, 53.9%, 54.9%, and 49.0%, respectively. Moreover, ROC analysis of FeNO combined with small airway parameters (MMEF_75%/25%_, FEF_50%_, FEF_25%_, Fres, R5-R20, or X5) was performed to further enhance the value of differentiation CVA from TA (Figure 3). The AUC of FeNO combined with MMEF_75%/25%_ (combine1) was 0.912 (0.873–0.952), combined with FEF_50%_ (combine2) was 0.893 (0.849–0.936), combined with FEF_25%_ (combine3) was 0.914 (0.875–0.953), combined with Fres (combine4) was 0.742 (0.673–0.810), combined with R5-R20 (combine5) was 0.707 (0.637–0.777), and combined with X5 (combine6) was 0.711 (0.640–0.782). The AUC of the combines was significantly higher than that of FeNO alone or small airway parameters FEF25%, Fres, R5-R20, and X5 alone (*P* < 0.05, Table 4). It was worth noting that there was no statistically significant in AUC between the combines and MMEF_75%/25%_ (*P*=0.106, Table 4) or FEF_50%_ (*P*=0.085, Table 4), and the improvement was mainly in sensitivity rather than specificity.

## 4. Discussion

CVA was mainly characterized as spasm of the small airway, while the large airway was not significantly impaired. The airway inflammation of CVA was less severe than that of TA, and the lung function was manifested as changes in small airway ventilation function, while TA had varying degrees of obstruction in both large and small airways [20]. Early diagnosis and intervention are thought to be important for asthma control. In general, CVA can easily evolve into TA, and nearly 30% of patients with CVA are found to eventually develop TA, so CVA is considered to be a precursor of TA. The early diagnosis of CVA and the distinction between CVA and TA can help understand the progression of the disease and better control the disease. However, there are some limitations in the current diagnosis of CVA, and the guidelines recommend pulmonary function test and BHR test as the first-line detection [16]. However, BHR has a low utilization rate and is mostly distributed in the grade-A tertiary hospitals due to its particularity [21]. Moreover, the diagnosis based on treatment effects will lead to drug abuse and difficult diagnosis [22, 23]. In contrast, spirometry and FeNO tests are more feasible due to the advantages of safety, speed, and simplicity. In this study, FeNO and small airway function were taken as research objects to explore the diagnostic value of FENO combined with small airway indexes in distinguishing CVA and TA, so as to better understand the disease process.

FeNO can be used to assess chronic airway inflammation from central large airways to surrounding small airways [24]. In this study, it was found that FeNO levels were significantly higher in TA subjects than in CVA subjects, suggesting that TA subjects had a higher level of chronic airway inflammation than CVA subjects, which is consistent with the conclusions of previous studies [25]. The correlation analysis between FeNO and pulmonary function indexes showed that FeNO had no correlation with pulmonary function indexes. The results of this study and previous relevant studies [26] all indicated that FeNO and the severity of airflow obstruction reflected by pulmonary function were not parallel. FeNO and pulmonary function were used to evaluate asthma from the aspects of airway inflammation and airway ventilation function, respectively. In addition, the optimal diagnostic threshold of FeNO and the sensitivity and specificity of diagnosis were varied greatly in previous studies. Kowal et al. has reported that the cut-off value to distinguish a chronic cough with and without asthma was 40 ppb with sensitivity of 88.3% and specificity of 82.6% [27]. The research of Sato et al. showed that the optimal threshold of FeNO for the diagnosis of TA and CVA in patients was 38.8 ppb with the sensitivity of 79.2% and the specificity of 91.3% [28]. Yi et al. showed that the best cut-off point of FeNO was 33.5 ppb with sensitivity of 69.6% and specificity of 85.1% [29], and Maniscalco et al. reported the optimal cut-off value was 33.0 ppb with sensitivity of 92% and specificity of 88% [30]. Our study indicated a cut-off value of 48.5 ppb with sensitivity of 90.4% and specificity of 42.2% for differentiation between CVA and TA, which obtained a higher optimal cut-off point than the previous studies. In general, FeNO had a good clinical application value and can be used to distinguish CVA and TA.

Small airway plays an important role in asthma, and there are small airway lesions in patients with asthma [31, 32]. Similar to large airway inflammation, small airway inflammation also leads to airway wall thickening, airway narrowing, and hyperresponsiveness, which leads to poor control and frequent exacerbation of asthma [33]. In our research, the pulmonary ventilation function indexes of TA subjects were significantly lower than those of CVA subjects, indicating that the pulmonary function impairment of TA subjects was more obvious than that of CVA subjects. In addition, PD20 value in CVA subjects was higher than that in TA subjects, supporting the idea that airway reactivity of CVA was lower than that of TA [25, 26]. The small airway flow velocity measurement indexes MMEF_75%/25%_, FEF_50%_, FEF_25%_, and small airway resistance measurement indexes Z5, Fres, R5-R20, X5, and X20 in the CVA subjects were also significantly different from those in the TA subjects, suggesting that TA subjects not only suffer from impairment of large airway function, but also small airway function. Compared with the CVA group, TA subjects showed more severe airway remodeling degree, worse lung function and higher airway resistance. This is consistent with the results of bronchial mucosal biopsy on asthma patients [34]. In addition, correlation analysis between small airway function indicators and other pulmonary function indicators proved that small airway function indicators including MMEF_75%/25%_, FEF_50%_, and FEF_25%_ were significantly positively correlated with FEV1/FVC both in CVA and TA subjects, indicating that small airway function was closely correlated to pulmonary expiratory function and can be used to evaluate asthma as airway ventilation function. However, there was no correlation between small airway function and FENO in our research. Although some studies believed that small airway function in asthma subjects was affected by airway inflammation [14], the results of this study indicated that small airway function could not be used to represent airway inflammation. Therefore, small airway function and FeNO should be combined to evaluate asthma from the perspective of pulmonary expiratory function and airway inflammation.

Our study found that both small airway function indicators and FeNO had good clinical diagnostic value for distinguishing CVA from TA. In the study of small airway function, MMEF_75%/25%_, FEF_50%_, and FEF_25%_ are the most commonly used flow velocity measurement methods, and impulse oscillometry is the most commonly used resistance measurement method [35]. Previous studies believed that impulse oscillometry indicators were more sensitive to reflect small airway dysfunction than conventional pulmonary ventilation function indicators [36], but in this study, MMEF_75%/25%_, FEF_50%,_ and FEF_25%_ were more sensitive to distinguish CVA from TA than impulse oscillometry indicators. Previous studies have shown that FeNO combined with impulse oscillometry indicators are helpful in diagnosis of small airway dysfunction with high sensitivity and specificity [37]. Therefore, ROC analysis of FeNO combined with small airway parameters was performed to further enhance the value of differentiation CVA from TA. The AUC of FeNO combined with small airway function indexes FEF_25%_, Fres, R5-R20, and X5 was significantly higher than the AUCs of individual indicators, while the effect of combined indicators on AUC of MMEF_75%/25%_ and FEF_50%_ was not statistically significant, and the improvement was mainly in sensitivity. But in general, the diagnostic values of combined indicators were significantly superior to that of a single parameter. Airway hyperreactivity and small airway dysfunction are common characteristics of CVA and TA. The airway inflammation of CVA subjects is weaker than that of TA, and the lung function damage is less than that of TA, which indicates that CVA is the early stage of TA [38]. Since the combined indicators could significantly improve the diagnostic value, they may be helpful in the identification of CVA from TA to understand the disease better.

In conclusion, FeNO and small airway function indicators can be used to assess asthma from the two directions of airway inflammation and airway ventilation function respectively, which may be useful for distinguishing CVA from TA. The combination of FeNO and small airway function indicators reflected the pathophysiological characteristics of asthma more comprehensively, and further improved the diagnostic value of distinguishing CVA from TA.

## Figures and Tables

**Table 1:** The demographic characteristics of TA and CVA subjects.

	TA (*n* = 102)	CVA (*n* = 104)	*t*/*χ*^2^	*P*
Gender				
Male	62 (60.8%)	48 (46.2%)	4.429	0.035^*∗a*^
Female	40 (39.2%)	56 (53.8%)		

Age (year)	58.08 ± 11.84	44.51 ± 13.54	7.61	<0.001^*∗∗∗b*^
<45	12 (11.8%)	48 (46.2%)	29.501	<0.001^*∗∗∗a*^
>45	90 (88.2%)	56 (53.8%)		

BMI (kg/m^2^)	25.05 ± 3.12	25.32 ± 3.53	0.58	0.56^b^

CVA, cough-variant asthma; TA, typical asthma. Data were shown as mean ± standard deviation. ^a^: *χ*^2^ test for differences between patients. ^b^: two-tailed Student's *T*-test between patients.

**Table 2:** Pulmonary function test results of subjects with TA and CVA.

	TA (*n* = 102)	CVA (*n* = 104)	*P*
FEV_1_% (%)	78.43 (19)	101.19 (14)	<0.001^*∗∗∗*^
FVC (%)	96.57 ± 14.01	106.44 ± 10.87	<0.001^*∗∗∗*^
FEV_1_/FVC	70.29 ± 6.56	78.94 ± 6.40	<0.001^∗∗∗^
FEV_1_/VCmax	68.44 (9)	98.81 (9)	<0.001^*∗∗∗*^
PEF (%)	86.09 ± 18.75	99.89 ± 18.01	<0.001^*∗∗∗*^
MMEF_75%/25%_ (%)	40.10 (17)	74.40 (29)	<0.001^*∗∗∗*^
FEF_50%_ (%)	44.20 (21)	80.82 (30)	<0.001^*∗∗∗*^
FEF_25%_ (%)	32.42 (17)	61.89 (28)	<0.001^*∗∗∗*^
PD20 (µmoL)	0.89 (2)	2.52 (4)	<0.001^*∗∗∗*^
D_L_CO	82.86 ± 19.72	90.14 ± 17.11	0.005^*∗*^
RV/TLC	112.35 (16)	103.25 (13)	<0.001^*∗∗∗*^
TLC	91.52 ± 11.27	96.75 ± 9.16	<0.001^*∗∗∗*^
RV	101 (25)	102.2 (19)	0.451
Z_5_ (pred)	1.23 (0)	1.09 (0)	0.014^*∗∗*^
Fres (Hz)	16.88 (7)	13.47 (5)	<0.001^∗∗∗^
R5 (pred)	1.17 (1)	1.08 (0)	0.640
R20 (pred)	1.08 (0)	1.11 (0)	0.268
R5-R20 (kPa·L^−1^·s^−1^)	0.45 (1)	0.34 (0)	<0.001^*∗∗∗*^
X5	−1.30 (1)	−1.14 (1)	<0.001^*∗∗∗*^
X20	0.25 (1)	0.62 (0)	<0.001^*∗∗∗*^
FeNO (ppb)	45 (37)	27 (18)	0.007^*∗*^

CVA, cough-variant asthma; TA, typical asthma, FeNO, fractional exhaled nitric oxide; PEF, peak expiratory flow; MMEF_75%/25%_, forced expiratory flow between 25% and 75%; FEF_50%_, forced expiratory flow at 50% of the FVC; FEF_25%_, forced expiratory flow at 25% of the FVC; TLC, total lung capacity; RV, residual volume. Data were expressed as mean ± standard deviation or median (interquartile range). ^*∗*^*P* < 0.05; ^*∗∗*^0.001 < *P* < 0.005; ^*∗∗∗*^*P* < 0.001.

**Table 3:** Correlations between FeNO or small airways function and other parameters.

	Single correlation		Multiple regression analysis	
a. FeNO				
TA	*r*	*P*	*B*	*P*
Male/female	−0.132	0.186	−0.204	0.099
Age	−0.016	0.877	0.001	0.992
BMI	−0.051	0.611	0.045	0.704
FEV_1_/FVC	−0.140	0.159	0.047	0.694
PEF	−0.005	0.96	−0.096	0.433
PD20 (µmoL)	−0.023	0.817	−0.096	0.384
D_L_CO	0.014	0.890	−0.095	0.395
RV/TLC	−0.030	0.763	0.079	0.483
CVA	*r*	*P*	*B*	*P*
Male/female	−0.131	0.186	−0.170	0.108
Age	0.044	0.656	−0.139	0.219
BMI	−0.080	0.422	−0.197	0.055
FEV_1_/FVC	−0.153	0.122	−0.067	0.586
PEF	−0.047	0.634	0.021	0.861
PD20 (µmoL)	0.003	0.976	−0.012	0.906
D_L_CO	0.082	0.410	−0.022	0.834
RV/TLC	0.129	0.191	0.159	0.140

b. MMEF_75%/25%_				
TA	*r*	*P*	*B*	*P*
Male/female	−0.158	0.112	−0.199	0.005^*∗∗*^
Age	−0.232	0.019^*∗*^	−0.078	0.216
BMI	0.050	0.615	−0.226	0.001^*∗∗∗*^
FEV_1_/FVC	0.699	<0.001^*∗∗∗*^	0.635	<0.001^*∗∗∗*^
PEF	0.500	<0.001^*∗∗∗*^	0.157	0.025^*∗*^
PD20 (µmoL)	0.093	0.351	-0.025	0.683
D_L_CO	0.264	0.007^*∗∗*^	0.200	0.002^*∗∗*^
RV/TLC	−0.375	<0.001^*∗∗∗*^	0.231	<0.001^*∗∗∗*^
FeNO	−0.075	0.454	−0.113	0.053
CVA	*r*	*P*	*B*	*P*
Male/female	−0.107	0.278	−0.173	<0.001^*∗∗∗*^
Age	−0.278	0.004^*∗∗*^	0.051	0.203
BMI	0.048	0.630	0.008	0.825
FEV_1_/FVC	0.905	<0.001^*∗∗∗*^	0.881	<0.001^*∗∗∗*^
PEF	0.431	<0.001^*∗∗∗*^	0.105	0.015^*∗*^
PD20 (µmoL)	0.197	0.045^*∗*^	0.025	0.493
D_L_CO	0.263	0.007^*∗∗*^	0.019	0.613
RV/TLC	−0.209	0.034^*∗*^	−0.092	0.017^*∗*^
FeNO	−0.082	0.409	0.029	0.427

c. FEF_50%_				
TA	*r*	*P*	*B*	*P*
Male/female	−0.099	0.323	−0.174	0.006^*∗∗*^
Age	−0.268	0.006^*∗∗*^	−0.090	0.114
BMI	0.053	0.599	−0.227	<0.001^*∗∗∗*^
FEV_1_/FVC	0.810	<0.001^*∗∗∗*^	0.717	<0.001^*∗∗∗*^
PEF	0.532	<0.001^*∗∗∗*^	0.120	0.058
PD20 (µmoL)	0.100	0.318	0.012	0.831
D_L_CO	0.207	0.037^*∗*^	0.179	0.002^*∗∗*^
RV/TLC	−0.351	<0.001^*∗∗∗*^	−0.205	0.001^*∗∗∗*^
FeNO	−0.101	0.314	−0.084	0.114
CVA	*r*	*P*	*B*	*P*
Male/female	−0.057	0.564	−0.096	0.068
Age	−0.229	0.019^*∗*^	0.088	0.114
BMI	0.066	0.503	0.036	0.469
FEV_1_/FVC	0.861	<0.001^*∗∗∗*^	0.828	<0.001^*∗∗∗*^
PEF	0.460	<0.001^*∗∗∗*^	0.101	0.091
PD20 (µmoL)	0.208	0.034^*∗*^	0.021	0.675
D_L_CO	0.202	0.039^*∗*^	0.008	0.876
RV/TLC	−0.229	0.019^*∗*^	−0.158	0.003^*∗∗*^
FeNO	−0.127	0.200	0.007	0.887

d. FEF25%				
TA	*r*	*P*	*B*	*P*
Male/female	−0.157	0.115	−0.273	0.003^*∗∗*^
Age	−0.133	0.182	−0.101	0.221
BMI	−0.054	0.588	−0.363	<0.001^*∗∗∗*^
FEV_1_/FVC	0.437	<0.001^*∗∗∗*^	0.467	<0.001^*∗∗∗*^
PEF	0.352	<0.001^*∗∗∗*^	0.107	0.239
PD20 (µmoL)	−0.051	0.609	−0.121	0.139
D_L_CO	0.314	0.001^*∗∗∗*^	0.232	0.006^*∗∗*^
RV/TLC	−0.307	0.002^*∗∗*^	−0.194	0.020^*∗*^
FeNO	−0.048	0.631	−0.150	0.051
CVA	*r*	*P*	*B*	*P*
Male/female	−0.125	0.207	−0.233	<0.001^*∗∗∗*^
Age	−0.401	<0.001^*∗∗∗*^	0.010	0.836
BMI	−0.009	0.926	−0.087	0.052
FEV_1_/FVC	0.900	<0.001^*∗∗∗*^	0.888	<0.001^*∗∗∗*^
PEF	0.262	0.007^*∗∗*^	−0.003	0.950
PD20 (µmoL)	0.198	0.044^*∗*^	0.030	0.504
D_L_CO	0.288	0.003^*∗∗*^	0.033	0.465
RV/TLC	−0.116	0.240	0.031	0.510
FeNO	−0.080	0.422	0.038	0.390

^*∗*^*P* < 0.05; ^*∗∗*^0.001 < *P* < 0.005; ^*∗∗∗*^*P* < 0.001.

**Table 4:** Optimal cut-off values for the prediction of CVA.

Parameter	AUC (95% CI)	*P*	Cut-off	Sensitivity (%)	Specificity (%)
FeNO	0.660 (0.585–0.735)	<0.001	48.50	90.4	42.2
MMEF_75%/25%_	0.895 (0.850–0.939)	<0.001	60.02	78.8	87.3
FEF_50%_	0.873 (0.825–0.921)	<0.001	63.46	80.8	82.4
FEF_25%_	0.898 (0.854–0.942)	<0.001	45.26	84.6	85.3
Fres	0.695 (0.623–0.767)	<0.001	16.63	81.7	53.9
R5-R20	0.650 (0.575–0.725)	<0.001	0.38	74.0	54.9
X5	0.645 (0.570–0.720)	<0.001	−1.32	74.0	49.0
Combine1	0.912 (0.873–0.952)^*∗∗∗*^	<0.001	—	83.7	87.3
Combine2	0.893 (0.849–0.936)^*∗∗∗*^	<0.001	—	86.5	83.4
Combine3	0.914 (0.875–0.953)^*∗∗∗*##^	<0.001	—	84.7	90.2
Combine4	0.742 (0.673–0.810)^*∗*$^	<0.001	—	77.0	65.8
Combine5	0.707 (0.637–0.777)^*∗*&^	<0.001	—	71.2	61.8
Combine6	0.711 (0.640–0.782)^*∗aa*^	<0.001	—	80.8	57.9

Combine 1: FeNO + MMEF_75%/25%_; Combine 2: FeNO + FEF_50%_; Combine 3: FeNO + FEF_25%_; Combine 4: FeNO + Fres; Combine 5: FeNO + R5-R20; Combine 6: FeNO + X5. ^*∗*^*P* < 0.05, ^*∗∗*^0.001 < *P* < 0.005, ^*∗∗∗*^*P* < 0.001, compared with FeNO; #*P* < 0.05; ##0.001 < *P* < 0.005, compared with FEF_25%_; $*P* < 0.05, compared with Fres; and *P* < 0.05, compared with R5-R20; ^aa^0.001 < *P* < 0.005, compared with X5.

## Data Availability

The datasets used and/or analyzed during the current study are available from the corresponding author on reasonable request.
